# Abatacept used in combination with non-methotrexate disease-modifying antirheumatic drugs: a descriptive analysis of data from interventional trials and the real-world setting

**DOI:** 10.1186/s13075-017-1488-5

**Published:** 2018-01-02

**Authors:** Rieke Alten, Harald Burkhardt, Eugen Feist, Klaus Krüger, Juergen Rech, Andrea Rubbert-Roth, Reinhard E. Voll, Yedid Elbez, Christiane Rauch

**Affiliations:** 1 Department of Internal Medicine, Rheumatology, Clinical Immunology and Osteology, Schlosspark-Klinik University Medicine Berlin, Heubnerweg 2, 14059 Berlin, Germany; 2 University Hospital Frankfurt, Goethe University, Heubnerweg 2, 14059 Frankfurt am Main, Germany; 3 Charité Universitaetsmedizin Berlin, Berlin, Germany; 4 Praxiszentrum St. Bonifatius, Munich, Germany; 5 University Clinic Erlangen, Erlangen, Germany; 6 Med Clinic I, University of Cologne, Cologne, Germany; 7 Medical Center—University of Freiburg, Faculty of Medicine, University of Freiburg, Freiburg, Germany; 8 Excelya, Boulogne-Billancourt, France; 9 Bristol-Myers Squibb, Munich, Germany

**Keywords:** Abatacept, Conventional synthetic disease-modifying antirheumatic drug, Azathioprine, Hydroxychloroquine, Leflunomide, Methotrexate, Sulfasalazine, Rheumatoid arthritis

## Abstract

**Background:**

Methotrexate (MTX) remains the anchor drug in rheumatoid arthritis (RA) treatment, but is poorly tolerated or contraindicated in some patients. There is a wealth of data supporting the use of abatacept in combination with MTX, but data on alternative conventional synthetic disease-modifying antirheumatic drug (csDMARD) combinations with abatacept are scarce.

**Methods:**

In this post-hoc exploratory analysis, efficacy and safety data were extracted from abatacept RA studies in which combination with csDMARDs other than MTX was permitted: three interventional trials (ATTAIN, ASSURE, and ARRIVE) and one real-world study (ACTION). Patients with moderate-to-severe RA received abatacept in combination with MTX, hydroxychloroquine, sulfasalazine, azathioprine, or leflunomide for 6 months to 2 years according to the study design. Change from baseline in physical function (Health Assessment Questionnaire—Disability Index (HAQ-DI); all studies) and 28-joint Disease Activity Score (C-reactive protein) (DAS28 (CRP); ATTAIN, ARRIVE, and ACTION), American College of Rheumatology response rates (ATTAIN), and safety were assessed for individual and pooled csDMARD combinations for each trial. A meta-analysis was also performed on pooled data for HAQ-DI and DAS28 (CRP) across interventional trials.

**Results:**

Across all four studies, 731 patients received abatacept plus one non-MTX csDMARD (hydroxychloroquine *n* = 152; sulfasalazine *n* = 123; azathioprine *n* = 59; and leflunomide *n* = 397) and 2382 patients received abatacept plus MTX. Mean changes from baseline in HAQ-DI scores for abatacept plus MTX (all csDMARDs pooled) vs abatacept plus a non-MTX csDMARD were –0.54 vs –0.44 (ATTAIN), –0.43 vs –0.43 (ASSURE), and –0.39 vs –0.36 (ARRIVE). Mean changes from baseline in DAS28 (CRP) and ACR response rates were also similar with abatacept plus MTX or non-MTX csDMARDs. Data for individual non-MTX csDMARDs (pooled across studies) and real-world data were consistent with these findings. Rates of treatment-related adverse events and serious adverse events, respectively, for abatacept plus one non-MTX csDMARD vs abatacept plus MTX were 35.7% vs 41.7% and 2.4% vs 2.3% (ATTAIN), 58.0% vs 55.9% and 4.2% vs 1.7% (ASSURE), and 38.1% vs 44.3% and 0.6% vs 2.9% (ARRIVE).

**Conclusions:**

Abatacept in combination with non-MTX csDMARDs is clinically effective and well tolerated in patients with moderate-to-severe RA, providing similar benefits to those seen with abatacept plus MTX.

**Trial registration:**

ClinicalTrials.gov NCT00048581. Registered 2 November 2002.

ClinicalTrials.gov NCT00048932. Registered 11 November 2002.

ClinicalTrials.gov NCT00124982. Registered 30 June 2005.

ClinicalTrials.gov NCT02109666. Registered 8 April 2014.

**Electronic supplementary material:**

The online version of this article (doi:10.1186/s13075-017-1488-5) contains supplementary material, which is available to authorized users.

## Background

Disease-modifying antirheumatic drugs (DMARDs) are the mainstay of treatment for patients with rheumatoid arthritis (RA). Both American College of Rheumatology (ACR) and European League Against Rheumatism (EULAR) guidelines recommend methotrexate (MTX) as the anchor drug in the first-line treatment of early or established RA [1–3], unless contraindicated or the patient is intolerant. In cases of persistent moderate/high disease activity despite MTX optimization and/or poor prognostic factors, combination therapy comprising MTX with additional conventional synthetic (cs) or biologic (b) DMARDs can be considered in line with a treat-to-target strategy [3–5].

For patients in whom MTX is contraindicated (e.g. in pregnancy and in those with renal or hepatic disease) or who have intolerance to MTX, alternative therapeutic options are needed. In such cases, csDMARD therapy with sulfasalazine (SSZ) or leflunomide (LEF) alone or in combination is recommended [1–3]. When poor prognostic factors (e.g. seropositivity, erosions) are present or disease control is poor, addition of a bDMARD or a targeted synthetic DMARD should be considered [1–4]. While there is a wealth of data to support the use of bDMARDs in combination with MTX [4], more data are needed on the efficacy and safety of bDMARDs in combination with csDMARDs other than MTX. Nonetheless, studies have shown some non-MTX csDMARDs are well tolerated and have similar efficacy to MTX in combination with tumour necrosis factor-alpha inhibitors (TNFi) [6, 7], the interleukin-6 inhibitor tocilizumab [8], or the B-cell-targeting agent rituximab [9, 10]. Indeed, the current EULAR guidelines recommend combining bDMARDs and targeted synthetic DMARDs with MTX or other csDMARDs if MTX cannot be used [3]. However, further studies are needed to support the use of bDMARDs with agents other than MTX.

Abatacept is a fusion protein comprising the cytotoxic T-lymphocyte-associated antigen-4 and the Fc region of human immunoglobulin G1 that binds to CD80/CD86 on antigen-presenting cells, thereby selectively modulating T-cell co-stimulation [11]. In Europe, abatacept in combination with MTX is indicated for the treatment of adult patients with moderate-to-severe, active RA and a prior inadequate response to one or more DMARDs including MTX and/or a TNFi and, in a recent update, in MTX-naïve patients who have highly active and progressive disease [12, 13].

The efficacy of abatacept in combination with MTX in treating the signs and symptoms of both early [14–16] and established [17–19] RA, reducing progression of joint damage and improving physical function, is well reported. In contrast, data regarding abatacept efficacy in combination with csDMARDs other than MTX are scarce; however, no major safety issues have been identified with abatacept in combination with SSZ, hydroxychloroquine (HCQ), or LEF [12]. In certain abatacept trials [17, 20–22], patients could receive abatacept in combination with csDMARDs other than MTX, providing the opportunity for further analysis of abatacept and csDMARD combination therapy. The aim of this post-hoc exploratory analysis was to report the efficacy and safety of abatacept in combination with csDMARDs other than MTX, based on available data from interventional trials and the real-world setting.

## Methods

### Study designs and patient populations

Efficacy and safety data for patients with moderate-to-severe RA who received abatacept or placebo in combination with MTX, HCQ, SSZ, azathioprine (AZA), or LEF were extracted from the ATTAIN (ClinicalTrials.gov NCT00048581), ASSURE (ClinicalTrials.gov NCT00048932), ARRIVE (ClinicalTrials.gov NCT00124982), and ACTION (ClinicalTrials.gov NCT02109666) studies. Details of the designs, patient populations, and primary results for these studies have been published previously [17, 21, 22]. Briefly, ATTAIN was a 6-month, randomized, double-blind, placebo-controlled study to assess the efficacy and safety of abatacept added to background DMARDs in patients with active RA who had an inadequate response to TNFi therapy [17]. ASSURE was a 1-year, multinational, multicentre, randomized, double-blind, two-arm, parallel-dosing trial to assess the safety of abatacept in patients with active RA during 1 year of abatacept treatment added to background therapy with ≥ 1 csDMARD or bDMARD [22]. ARRIVE was an international, 6-month, open-label trial in patients with active RA who had an inadequate response to TNFi therapy for ≥ 3 months and 28-joint Disease Activity Score (C-reactive protein) (DAS28 (CRP)) ≥ 5.1 and were switched to abatacept directly from their TNFi or after completing washout of TNFi therapy within 2 months of screening [21]; background csDMARDs were continued after the switch. ACTION was a prospective, observational study in patients with RA who initiated intravenous abatacept in Europe and Canada between May 2008 and January 2013 to assess retention, effectiveness, safety, and tolerability of abatacept in a real-world setting. The population included patients enrolled in Cohort A (May 2008–December 2010) who were naïve to bDMARDs or who had previously failed treatment with ≥ 1 bDMARD—most (1009/1131 (89.2%)) had experienced prior biologic failure [13, 20].

### Outcomes and assessments in the present analysis

ACR response rates reflect improvements in the signs and symptoms of RA; specifically, tender and swollen joints, and three of the five remaining core data set measures: patient global assessment of pain, patient global assessment of disease activity, physician global assessment of disease activity, patient assessment of physical function (Health Assessment Questionnaire—Disability Index (HAQ-DI)), and an acute-phase reactant value, CRP. In the ATTAIN study, ACR response rates of ≥ 20%, ≥ 50%, and ≥ 70% improvement (ACR20, ACR50, and ACR70), the mean change from baseline in DAS28 (CRP), the HAQ-DI score, and safety were assessed at 6 months [17]. In the ASSURE study, the mean change from baseline in HAQ-DI score was measured at 6 and 12 months, and the safety of abatacept added to a background of ≥ 1 csDMARD was assessed [22]. In the ARRIVE study, DAS28 (CRP) and HAQ-DI score were determined and safety was assessed in all patients who received ≥ 1 dose of study drug at 6 months [21]. In the ACTION study, mean change from baseline in DAS28 (CRP) and HAQ-DI score was determined at 6, 12, and 24 months, and safety was assessed in accordance with local regulations [20].

Safety data were summarized by treatment (individual and pooled for all non-MTX csDMARDs) for the ATTAIN, ASSURE, and ARRIVE studies. In the ACTION study, safety data for individual csDMARDs were not assessed; therefore, the safety profiles of abatacept in combination with MTX or other csDMARDs could not be compared.

### Analyses

Available baseline disease characteristics and demographics were reported for each treatment (abatacept or placebo plus MTX or other csDMARD) using descriptive statistics, including sample size, proportions or means, and standard deviations (SDs).

Comparison of efficacy and safety outcomes for abatacept vs placebo could not be performed for combination therapy with any csDMARD other than with MTX due to low numbers of patients who received these therapies. Furthermore, formal comparisons between abatacept plus MTX vs a non-MTX csDMARD could not be conducted due to large imbalances in patient numbers between treatment groups and very small patient numbers for some csDMARDs. Therefore, only outcomes for abatacept plus MTX or other csDMARD were reported, with results analysed descriptively.

Efficacy outcomes were investigated at specific time points (6, 12, and/or 24 months) according to the follow-up available in each study. For the interventional studies, within-trial efficacy data were pooled and reported for abatacept plus any one non-MTX csDMARD for each available outcome from that trial. Efficacy data from the ACTION study were also reported for abatacept plus MTX or abatacept plus any non-MTX csDMARD given as first-line or later-line therapy. In addition, efficacy results for abatacept with MTX or one other csDMARD were pooled across studies when an outcome was included in more than one interventional study (ATTAIN, ASSURE, or ARRIVE). Integrated HAQ-DI data across the three interventional studies (ATTAIN, ASSURE, and ARRIVE) and DAS28 (CRP) data across two studies (ATTAIN and ARRIVE) were pooled for individual csDMARDs. The pooled variance method was used to estimate means and proportions with 95% confidence intervals (CIs) combined from multiple studies, in order to shorten the CI widths and consequently increase statistical precision due to the greater number of patients when combining multiple studies.

Safety outcomes were assessed at 6 months in the ATTAIN and ARRIVE studies and at 12 months in the ASSURE study, representing the short-term/double-blind periods of these studies.

## Results

### Patient population

Across all four studies, 2382 patients included in the analysis received abatacept plus MTX and 731 received abatacept plus one non-MTX csDMARD (HCQ *n* = 152; SSZ *n* = 123; AZA *n* = 59; LEF *n* = 397) (Table 1). Baseline characteristics for patients, by csDMARD subgroup, are presented in Table 2; there were no major differences between groups or studies. The mean dose of each csDMARD used in combination with abatacept was similar across the three interventional trials (Table 3). In the ACTION study, csDMARDs were used according to the label.

**Table 1:** Numbers of patients participating in the ATTAIN, ASSURE, ARRIVE, and ACTION studies who were taking one csDMARD in combination with abatacept or placebo

	Number of patients				
	ATTAIN	ASSURE	ARRIVE	ACTION	Total
ABA + MTX	175	535	490	1182	2382
PBO + MTX	89	243	–	–	332
ABA + HCQ	11	35	49	57	152
PBO + HCQ	2	33	–	–	35
ABA + SSZ	10	36	32	45	123
PBO + SSZ	4	10	–	–	14
ABA + AZA	4	10	30	15	59
PBO + AZA	1	9	–	–	10
ABA + LEF	17	62	65	253	397
PBO + LEF	7	29	–	–	36

*ABA* abatacept, *AZA* azathioprine, *csDMARD* conventional synthetic disease-modifying antirheumatic drug, *HCQ* hydroxychloroquine, *LEF* leflunomide, *MTX* methotrexate, *PBO* placebo, *SSZ* sulfasalazine

**Table 2:** Baseline characteristics of patients participating in the ATTAIN, ASSURE, ARRIVE, and ACTION studies who were taking one csDMARD in combination with abatacept

	ABA + MTX	ABA + HCQ	ABA + SSZ	ABA + AZA	ABA + LEF
ATTAIN cohort	(*n* = 175)	(*n* = 11)	(*n* = 10)	(*n* = 4)	(*n* = 17)
Female, *n* (%)	140 (80)	8 (73)	7 (70)	3 (75)	14 (82)
Age (years)	52.85 (11.79)	52.91 (15.02)	55.10 (14.29)	63.50 (16.43)	50.76 (15.03)
DAS28 (ESR)	6.9 (1.03)	7.0 (0.73)	6.6 (0.75)	7.3 (1.19)	7.2 (1.08)
CDAI LDA/remission, *n* (%)	–	–	–	–	–
RF positive, *n* (%)	129 (74)	9 (82)	7 (70)	1 (25)	12 (71)
ACPA positive, *n* (%)	–	–	–	–	–
ASSURE cohort	(*n* = 535)	(*n* = 35)	(*n* = 36)	(*n* = 10)	(*n* = 62)
Female, *n* (%)	439 (82)	33 (94)	28 (78)	10 (100)	47 (76)
Age (years)	52.19 (12.11)	54.77 (11.13)	53.89 (12.47)	51.10 (6.90)	54.76 (10.42)
DAS28 (ESR)	–	–	–	–	–
CDAI LDA/remission, *n* (%)	–	–	–	–	–
RF positive, *n* (%)	–	–	–	–	–
ACPA positive, *n* (%)	–	–	–	–	–
ARRIVE cohort	(*n* = 490)	(*n* = 49)	(*n* = 32)	(*n* = 30)	(*n* = 65)
Female, *n* (%)	405 (83)	43 (88)	22 (69)	25 (83)	53 (82)
Age (years)	55.35 (12.32)	51.31 (11.54)	57.00 (13.69)	55.27 (11.30)	53.38 (11.4)
DAS28 (CRP)	6.2 (0.72)	6.3 (0.81)	6.2 (0.75)	6.1 (0.74)	6.1 (0.72)
CDAI LDA/remission, *n* (%)	–	–	–	–	–
RF positive, *n* (%)	296 (60)	31 (63)	20 (63)	23 (77)	34 (52)
ACPA positive, *n* (%)	–	–	–	–	–
ACTION cohort	(*n* = 1182)	(*n* = 57)	(*n* = 45)	(*n* = 15)	(*n* = 253)
Female, *n* (%)	926 (78)	45 (79)	35 (78)	9 (60)	204 (81)
Age (years)	57.48 (12.31)	58.16 (13.16)	57.76 (11.89)	58.80 (14.11)	56.10 (13.59)
DAS28 (ESR)	5.4 (1.25)	5.5 (1.19)	5.5 (1.32)	5.4 (1.44)	5.4 (1.37)
CDAI LDA/remission, *n* (%)	31 (3.1)	1 (2.2)	2 (4.7)	1 (8.3)	14 (6.6)
RF positive, *n*/*N* (%)	738/1027 (71.9)	33/49 (67.3)	27/37 (73.0)	7/11 (63.6)	142/205 (69.3)
ACPA positive, *n*/*N* (%)	667/981 (68.0)	28/40 (70.0)	27/38 (71.1)	7/10 (70.0)	116/183 (63.4)

Data are mean (standard deviation) unless otherwise indicated

*ABA* abatacept, *AZA* azathioprine, *ACPA* anti-citrullinated protein antibody, *CDAI* Clinical Disease Activity Index, *CRP* C-reactive protein, *csDMARD* conventional synthetic disease-modifying antirheumatic drug, *DAS28* Disease Activity Score in 28 joints, *ESR* erythrocyte sedimentation rate, *HCQ* hydroxychloroquine, *LDA* low disease activity, *LEF* leflunomide, *MTX* methotrexate, *RF* rheumatoid factor, *SSZ* sulfasalazine

**Table 3:** Mean daily dose of each csDMARD used in combination with abatacept in the ATTAIN, ASSURE, and ARRIVE studies

	Daily dose (mg)		
	ATTAIN	ASSURE	ARRIVE
Methotrexate	2.17	3.88	2.60
Hydroxychloroquine	364	323	383
Sulfasalazine	2597	2108	1866
Azathioprine	121	121	124
Leflunomide	18.2	19.9	19.4

No data are available on the dose of individual csDMARDs used in the ACTION study

*csDMARD* conventional synthetic disease-modifying antirheumatic drug

### Efficacy

**Fig. 1:**
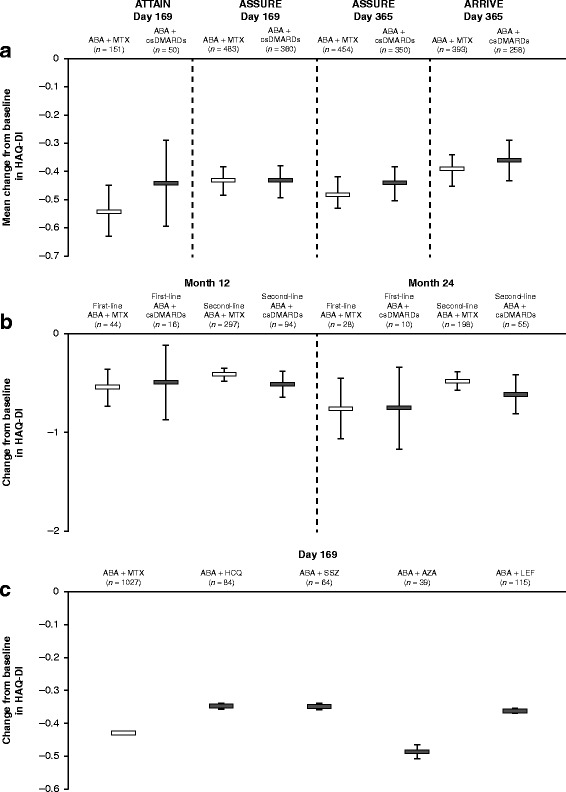
Mean change from baseline in HAQ-DI scores in response to abatacept administered in combination with one csDMARD (**a**) All non-MTX csDMARDs pooled in individual interventional studies. (**b**) Data from the observational ACTION study. (**c**) Pooled analysis from the ATTAIN, ASSURE, and ARRIVE studies for individual csDMARDs. Error bars show 95% CI. ABA abatacept, AZA azathioprine, csDMARD conventional synthetic disease-modifying antirheumatic drug, HAQ-DI Health Assessment Questionnaire—Disability Index, HCQ hydroxychloroquine, LEF leflunomide, MTX methotrexate, SSZ sulfasalazine

Mean change from baseline in HAQ-DI scores was available for all four studies. In patients treated with abatacept plus MTX vs abatacept plus any csDMARD other than MTX (pooled by study), the mean changes from baseline (95% CI) in HAQ-DI scores to month 6 were, respectively: –0.54 (–0.63, –0.45) vs –0.44 (–0.59, –0.29) (ATTAIN), –0.43 (–0.48, –0.38) vs –0.43 (–0.48, –0.37) (ASSURE), and –0.39 (–0.44, –0.33) vs –0.36 (–0.43, –0.29) (ARRIVE) (Fig. 1a). In the ACTION study, similar reductions in HAQ-DI scores were observed for abatacept administered in combination with MTX vs non-MTX csDMARDs, respectively, at 12 and 24 months (Fig. 1b); this finding was consistent, regardless of whether abatacept was given as first-line (12 months -0.54 vs –0.49; 24 months –0.76 vs –0.75) or second-line (12 months –0.41 vs –0.51; 24 months –0.48 vs –0.61) therapy (95% CIs overlapped; Fig. 1b).

In a pooled analysis of data from the three interventional studies (ATTAIN, ASSURE, and ARRIVE), patients treated with abatacept plus MTX had a mean change from baseline in HAQ-DI scores to month 6 of –0.43 (Fig. 1c); for individual non-MTX csDMARDs, this value ranged from –0.35 to –0.49 (Fig. 1c). When each csDMARD and interventional study were analysed separately, reductions in HAQ-DI scores with abatacept plus the non-MTX csDMARDs HCQ, SSZ, AZA, and LEF were similar to those observed with abatacept plus MTX, although 95% CIs were large for all treatments other than MTX (Additional file 1).

**Fig. 2:**
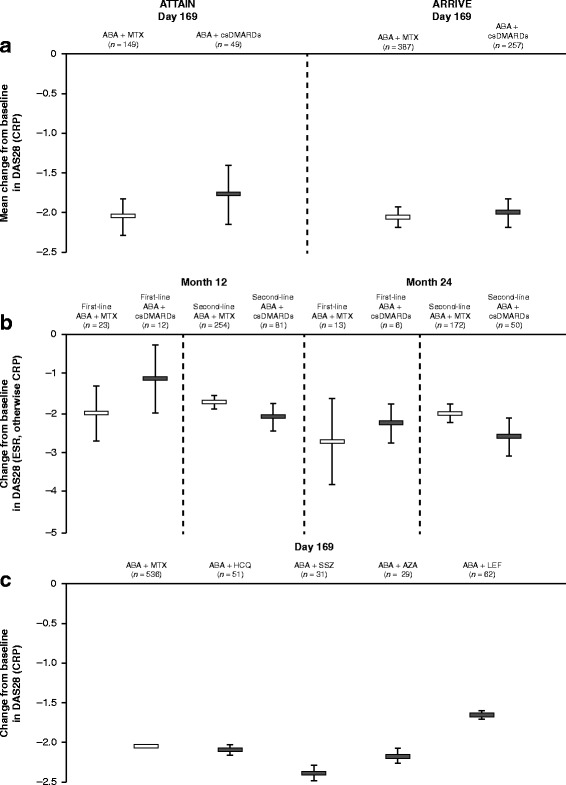
Mean change from baseline in DAS28 (CRP) in response to abatacept administered in combination with one csDMARD. (**a**) Pooled for all non-MTX csDMARDs in individual interventional studies. (**b**) Data from the observational ACTION study (DAS28 (ESR, otherwise CRP)). (**c**) Pooled analysis from the ATTAIN and ARRIVE studies. Error bars show 95% CI. ABA abatacept, AZA azathioprine, CRP C-reactive protein, csDMARD conventional synthetic disease-modifying antirheumatic drug, DAS28 28-joint Disease Activity Score, ESR erythrocyte sedimentation rate, HCQ hydroxychloroquine, LEF leflunomide, MTX methotrexate, SSZ sulfasalazine

Mean changes from baseline in DAS28 (CRP) were available for the ATTAIN, ARRIVE, and ACTION (DAS28 (erythrocyte sedimentation rate, otherwise CRP)) studies only. In the individual trials (pooled non-MTX csDMARD analysis), mean changes from baseline (95% CI) in DAS28 (CRP) for abatacept plus MTX vs abatacept plus any non-MTX DMARD were: –2.05 (–2.28, –1.81) vs –1.78 (–2.16, –1.40) (ATTAIN) and –2.03 (–2.16, –1.90) vs –2.00 (–2.18, –1.82) (ARRIVE) (Fig. 2a). In the ACTION study, similar reductions in DAS28 (CRP) were observed for abatacept administered in combination with MTX or non-MTX csDMARDs, respectively, at both 12 and 24 months, and irrespective of whether abatacept was given as first-line (12 months –2.03 vs –1.16; 24 months –2.74 vs –2.27) or second-line (12 months –1.73 vs –2.11; 24 months –2.02 vs –2.61) therapy (95% CIs overlapped; Fig. 2b).

In the pooled analysis of data from the ATTAIN and ARRIVE studies, the mean change from baseline in DAS28 (CRP) in patients treated with abatacept plus MTX was –2.04. In those treated with abatacept plus a csDMARD other than MTX, the mean change from baseline in DAS28 (CRP) ranged from –1.65 to –2.37 (Fig. 2c). Consistent with the effects of treatment on HAQ-DI, improvements in DAS28 (CRP) with abatacept plus individual csDMARDs were similar to those observed with abatacept plus MTX in the individual ATTAIN and ARRIVE studies (Additional file 2).

**Fig. 3:**
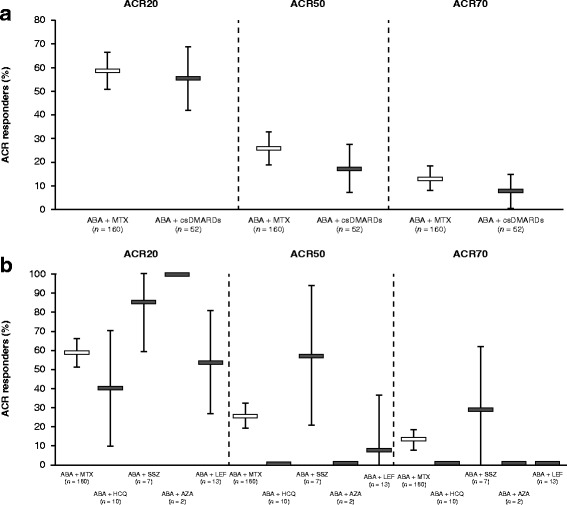
ACR response rates for abatacept administered in combination with (**a**) MTX vs any one non-MTX csDMARD (pooled analysis) and (**b**) individual csDMARDs at 6 months in the ATTAIN study. Excludes patients on multiple background csDMARDs. Error bars show 95% CI. ABA abatacept, ACR20/50/70, ≥ 20%/≥ 50%/≥ 70% improvement in American College of Rheumatology criteria, AZA azathioprine, csDMARD conventional synthetic disease-modifying antirheumatic drug, HCQ hydroxychloroquine, LEF leflunomide, MTX methotrexate, SSZ sulfasalazine

ACR20/50/70 response rates were assessed only in the ATTAIN study (Fig. 3). ACR20 response rates for abatacept plus MTX were 58.8% and for any other non-MTX csDMARD (pooled) were 55.8%. While there were numerical differences in ACR50 and ACR70 response rates for abatacept plus MTX vs abatacept plus any non-MTX csDMARD when data were pooled, 95% CIs overlapped between the two groups.

### Safety

Adverse events (AEs) in patients treated with abatacept administered in combination with MTX or with a non-MTX csDMARD (pooled data) for the ATTAIN, ASSURE, and ARRIVE studies are presented in Table 4. The incidence of any AE or serious AE (SAE), related AEs or SAEs, and discontinuation due to AEs or SAEs among patients who received abatacept plus MTX or those who received abatacept plus any non-MTX csDMARDs (pooled data) were similar within each study (Table 4).

**Table 4:** Safety of abatacept administered in combination with MTX or pooled data for other non-MTX csDMARDs in the ATTAIN, ASSURE, and ARRIVE studies

	ATTAIN		ASSURE		ARRIVE	
	ABA + MTX (*n* = 175)	ABA + all non-MTX csDMARDs (*n* = 42)	ABA + MTX (*n* = 535)	ABA + all non-MTX csDMARDs (*n* = 143)	ABA + MTX (*n* = 490)	ABA + all non-MTX csDMARDs (*n* = 176)
Deaths	0	1 (2.4)	3 (0.6)	1 (0.7)	1 (0.2)	0
SAEs	16 (9.1)	5 (11.9)	54 (10.1)	24 (16.8)	45 (9.2)	13 (7.4)
Related SAEs	4 (2.3)	1 (2.4)	9 (1.7)	6 (4.2)	14 (2.9)	1 (0.6)
Discontinuations due to SAEs	3 (1.7)	4 (9.5)	12 (2.2)	4 (2.8)	7 (1.4)	1 (0.6)
AEs	139 (79.4)	30 (71.4)	480 (89.7)	127 (88.8)	383 (78.2)	131 (74.4)
Related AEs	73 (41.7)	15 (35.7)	299 (55.9)	83 (58.0)	217 (44.3)	67 (38.1)
Discontinuations due to AEs	5 (2.9)	4 (9.5)	28 (5.2)	8 (5.6)	20 (4.1)	7 (4.0)
AEs of interest (system organ class)						
Infections and infestations	67 (38.3)	17 (40.5)	292 (54.6)	90 (62.9)	203 (41.4)	63 (35.8)
Skin and subcutaneous tissue disorders	27 (15.4)	7 (16.7)	133 (24.9)	37 (25.9)	61 (12.4)	24 (13.6)
Eye disorders	8 (4.6)	2 (4.8)	45 (8.4)	22 (15.4)	24 (4.9)	6 (3.4)
Cardiac disorders	7 (4.0)	5 (11.9)	28 (5.2)	11 (7.7)	16 (3.3)	5 (2.8)
Blood and lymphatic system disorders	2 (1.1)	3 (7.1)	28 (5.2)	10 (7.0)	10 (2.0)	3 (1.7)
Immune system disorders	2 (1.1)	1 (2.4)	13 (2.4)	5 (3.5)	7 (1.4)	4 (2.3)
Neoplasms (benign, malignant and unspecified)	3 (1.7)	2 (4.8)	20 (3.7)	3 (2.1)	6 (1.2)	2 (1.1)

All data are *n* (%)

Non-MTX csDMARDs include azathioprine, hydroxychloroquine, leflunomide, and sulfasalazine

*ABA* abatacept, *AE* adverse event, *csDMARD* conventional synthetic disease-modifying antirheumatic drug, *MTX* methotrexate, *SAE* serious adverse event

The rates of treatment-related AEs in patients who received abatacept plus MTX were 41.7% (ATTAIN), 55.9% (ASSURE), and 44.3% (ARRIVE), and in patients who received abatacept plus any non-MTX csDMARD were 35.7% (range 25.0–40.0%; ATTAIN), 58.0% (range 50.0–59.7%; ASSURE), and 38.1% (range 28.1–46.7%; ARRIVE; Table 4 and Additional file 3). The rates of treatment-related SAEs with abatacept plus MTX were 2.3% (ATTAIN), 1.7% (ASSURE), and 2.9% (ARRIVE), and with abatacept plus any non-MTX csDMARD were 2.4% (range 0.0–25.0%; ATTAIN), 4.2% (range 0.0–10.0%; ASSURE), and 0.6% (range 0–1.5%; ARRIVE). Rates of discontinuations due to AEs and SAEs are also presented in Table 4.

The number of deaths was very low across the studies. In patients receiving abatacept, there was one death in the ATTAIN study (abatacept plus SSZ), four in the ASSURE study (three with abatacept plus MTX and one with abatacept plus LEF), and one in the ARRIVE study (abatacept plus MTX). Infections and infestations were the most common AEs by system organ class. Rates were 38.3%, 54.6%, and 41.4%, respectively, for abatacept plus MTX in the ATTAIN, ASSURE, and ARRIVE studies, and 40.5% (range 10.0–64.7%) in ATTAIN, 62.9% (range 47.2–71.0%) in ASSURE, and 35.8% (range 25.0–46.7%) in ARRIVE for abatacept plus non-MTX csDMARDs (Table 4). For all safety outcomes, the broad ranges of AE rates for non-MTX csDMARDs reflected very low patient numbers in these groups.

## Discussion

This is the first analysis to explore available data for the safety and efficacy of abatacept in RA when administered in combination with csDMARDs other than MTX. These results from interventional clinical trials and the real-world setting suggest that a treatment regimen of abatacept plus csDMARDs other than MTX is well tolerated and clinically effective, improving physical function and ACR20 response rates, and reducing disease activity. Small patient numbers for most non-MTX csDMARDs and large imbalances in treatment group size prevented any statistical comparison of abatacept in combination with MTX vs other csDMARDs, or of abatacept vs placebo for non-MTX csDMARD combinations. When data for individual non-MTX csDMARDs within a single trial were pooled, the results suggested that clinical benefits of abatacept were similar whether combined with MTX or other csDMARDs. Further meta-analyses were performed where possible to address problems associated with small sample sizes and allow for analysis of individual non-MTX csDMARDs by combining data from different trials that employed the same endpoints. Overall, these data suggest that abatacept plus the non-MTX csDMARDs HCQ, SSZ, AZA, and LEF may be similarly effective to abatacept plus MTX. Real-world data from the ACTION study indicated that similar benefits were achieved for abatacept administered in combination with MTX vs other csDMARDs as either first-line or second-line therapy. Safety profiles were similar for abatacept administered in combination with MTX or any non-MTX csDMARDs combined (pooled data) in the ATTAIN, ASSURE, and ARRIVE studies.

MTX remains the anchor drug for the first-line treatment of RA [1–3]. However, many patients do not wish to continue MTX therapy due to a variety of reasons reflecting personal choice. For these patients who are unable or unwilling to take MTX, and in whom bDMARD therapy is indicated, more information is needed on the efficacy and safety of bDMARDs, including abatacept, in combination with csDMARDs other than MTX [1].

The efficacy and safety of abatacept plus MTX at various stages of RA is well established. In patients with early active RA who had received little or no prior MTX, abatacept plus MTX has been shown to be superior to MTX alone in achieving remission (DAS28 (CRP) < 2.6) and in maintenance of remission and structural benefits 6 months following withdrawal of all RA medication [16, 23]. Superior efficacy to placebo [24, 25] and comparable efficacy to adalimumab [26, 27] have been demonstrated in bDMARD-naïve MTX inadequate responders. Real-world data also indicate efficacy in bDMARD-naïve patients [20]. Efficacy following bDMARD failure has also been reported in the clinical trial [17] and real-world [13, 20] settings. Although some abatacept studies permitted the use of background csDMARDs other than MTX, these data have not been reported in detail previously.

The findings reported here are consistent with those for non-MTX csDMARDs in combination with other bDMARDs [6–10, 28]. Real-world data suggest that non-MTX csDMARDs are as effective as MTX in combination with TNFi [6, 7] or the interleukin-6 inhibitor tocilizumab [8]. Limited data are available on rituximab combined with non-MTX DMARDs; however, data extracted from 10 European registries (the CERERRA collaboration) and a small retrospective study suggested that LEF and MTX were similarly effective and well tolerated when administered in combination with rituximab [9, 10]. In addition, rituximab in combination with LEF was shown to demonstrate significant efficacy vs placebo plus LEF in a recent multicentre randomized controlled trial [28].

The present analysis also showed that abatacept administered in combination with csDMARDs was well tolerated. The rates of any AEs, SAEs, or discontinuations due to AEs or SAEs for abatacept plus non-MTX csDMARDs (pooled data) were similar to those observed with abatacept plus MTX. It is, however, difficult to make safety comparisons between MTX and all other csDMARDs given the low numbers of patients for some non-csDMARDs, in particular AZA and SSZ. Although MTX is recognized as having a good safety profile [3], it is contraindicated in patients with liver or kidney disease and in pregnancy, and intolerances do occur on a fairly frequent basis [29, 30]. A systematic literature review of 88 studies evaluating the long-term use of MTX in RA (mean duration 12.7 years) reported considerable variation in overall discontinuation rates due to AEs (range 10–37%), but these rates fell within a similar range to those observed with SSZ (17–52%) and HCQ (10–14%). Thus, as in our analysis, there seems to be little difference between overall safety of the different csDMARDs; although notably, only antimalarial agents and SSZ are considered to have an acceptable safety profile during pregnancy and when breastfeeding [31].

The limitations of this analysis must be considered when interpreting the data presented here. There are inherent limitations of post-hoc analyses, and this analysis was not designed or powered to detect differences between the treatment groups. Small patient numbers in some treatment groups limit the conclusions that can be drawn, and pooled analyses should be interpreted with caution. Although including ACTION data in the pooled analyses would have increased patient numbers further, it was not considered appropriate to pool data from the ACTION study and the interventional studies as ACTION was an observational study and drug administration by physician prescription may potentially have introduced biases that are removed by the randomization process. ACR response rate data were available from the ATTAIN trial only, with 52 patients receiving non-MTX csDMARDs. In addition, the mean dose of MTX used in the ATTAIN study (~15 mg/week) was rather low, which may have influenced efficacy. Safety of specific DMARDs in the real-world setting was also lacking as AEs in the ACTION study were not linked to the patient when recorded and, therefore, could not be attributed to a specific csDMARD. Despite these limitations, the current data add to existing knowledge regarding the safety and efficacy of csDMARDs other than MTX in combination with abatacept therapy.

## Conclusions

The results of this post-hoc analysis suggest that abatacept therapy in combination with csDMARDs other than MTX improves clinical outcomes and is well tolerated in patients with moderate-to-severe RA, achieving similar clinical benefits to those observed with abatacept plus MTX in both the first-line and the second-line setting.

## Additional files

Additional file 1: is a figure showing mean change from baseline in HAQ-DI scores in response to abatacept administered in combination with one csDMARD from the (a) ATTAIN, (b) ASSURE, and (c) ARRIVE studies (PDF 183 kb)

Additional file 2: is a figure showing mean change from baseline in DAS28 (CRP) at 6 months in response to abatacept administered in combination with one csDMARD from the (a) ATTAIN and (b) ARRIVE studies (PDF 101 kb)

Additional file 3: is a table presenting safety profile data of csDMARDs administered in combination with abatacept in the ATTAIN, ASSURE and ARRIVE studies (PDF 35 kb)

## Abbreviations

ABA: Abatacept
ACR20/50/70: ≥ 20%/≥ 50%/≥ 70% improvement in American College of Rheumatology criteria
AE: Adverse event
AZA: Azathioprine
bDMARD: Biologic disease-modifying antirheumatic drug
CI: Confidence interval
CRP: C-reactive protein
csDMARD: Conventional synthetic disease-modifying antirheumatic drug
DAS28: 28-joint Disease Activity Score
EULAR: European League Against Rheumatism
HAQ-DI: Health Assessment Questionnaire—Disability Index
HCQ: Hydroxychloroquine
LEF: Leflunomide
MTX: Methotrexate
RA: Rheumatoid arthritis
SAE: Serious adverse event
SSZ: Sulfasalazine
