# Supplementary material for: Abatacept used in combination with non-methotrexate disease-modifying antirheumatic drugs: a descriptive analysis of data from interventional trials and the real-world setting
Source: Arthritis Res Ther. 2018 Jan 2;20:1. doi: 10.1186/s13075-017-1488-5 (PMC5795278; doi:10.1186/s13075-017-1488-5)
Supplement: Supplementary file 1 — is a figure showing mean change from baseline in HAQ-DI scores in response to abatacept administered in combination with one csDMARD from the (a) ATTAIN, (b) ASSURE, and (c) ARRIVE studies (PDF 183 kb) [file 13075_2017_1488_MOESM1_ESM.pdf]

Additional file 1

Supplementary Fig 1. Mean change from baseline in HAQ-DI scores in response to abatacept administered in combination with one csDMARD from the **(a)** ATTAIN, **(b)** ASSURE, and **(c)** ARRIVE studies. Excludes patients on multiple background csDMARDs. Error bars show 95% CI. ABA, abatacept; AZA, azathioprine; CI, confidence interval; csDMARD, conventional synthetic disease-modifying antirheumatic drug; HAQ-DI, Health Assessment Questionnaire-Disability Index; HCQ, hydroxychloroquine; LEF, leflunomide; MTX, methotrexate; SSZ, sulfasalazine.

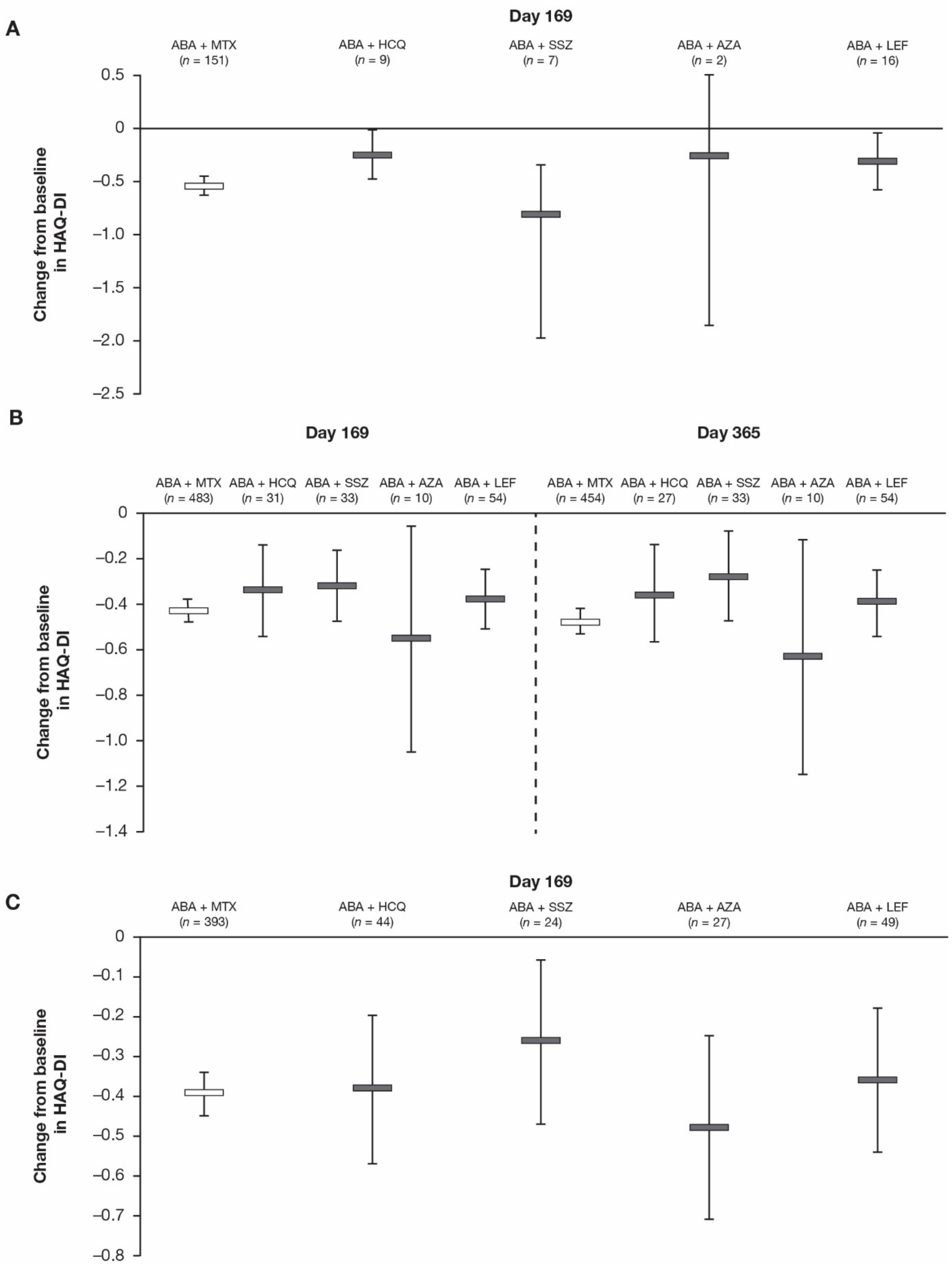

A
Day 169
ABA + MTX (n = 151)
ABA + HCQ (n = 9)
ABA + SSZ (n = 7)
ABA + AZA (n = 2)
ABA + LEF (n = 16)
Change from baseline in HAQ-DI
0.5
0
−0.5
−1.0
−1.5
−2.0
−2.5
B
Day 169
Day 365
ABA + MTX (n = 483)
ABA + HCQ (n = 31)
ABA + SSZ (n = 33)
ABA + AZA (n = 10)
ABA + LEF (n = 54)
ABA + MTX (n = 454)
ABA + HCQ (n = 27)
ABA + SSZ (n = 33)
ABA + AZA (n = 10)
ABA + LEF (n = 54)
Change from baseline in HAQ-DI
0
−0.2
−0.4
−0.6
−0.8
−1.0
−1.2
−1.4
C
Day 169
ABA + MTX (n = 393)
ABA + HCQ (n = 44)
ABA + SSZ (n = 24)
ABA + AZA (n = 27)
ABA + LEF (n = 49)
Change from baseline in HAQ-DI
0
−0.1
−0.2
−0.3
−0.4
−0.5
−0.6
−0.7
−0.8
