# Supplementary material for: Abatacept used in combination with non-methotrexate disease-modifying antirheumatic drugs: a descriptive analysis of data from interventional trials and the real-world setting
Source: Arthritis Res Ther. 2018 Jan 2;20:1. doi: 10.1186/s13075-017-1488-5 (PMC5795278; doi:10.1186/s13075-017-1488-5)

Additional file 2

Supplementary Fig 2. Mean change from baseline in DAS28 (CRP) at 6 months in response to abatacept administered in combination with one csDMARD from the **(a)** ATTAIN and **(b)** ARRIVE studies. Excludes patients on multiple background csDMARDs. Error bars show 95% CI. ABA, abatacept; AZA, azathioprine; CI, confidence interval; csDMARD, conventional synthetic disease-modifying antirheumatic drug; DAS28, 28-joint Disease Activity Score; CRP, C-reactive protein; HCQ, hydroxychloroquine; LEF, leflunomide; MTX, methotrexate; SSZ, sulfasalazine.

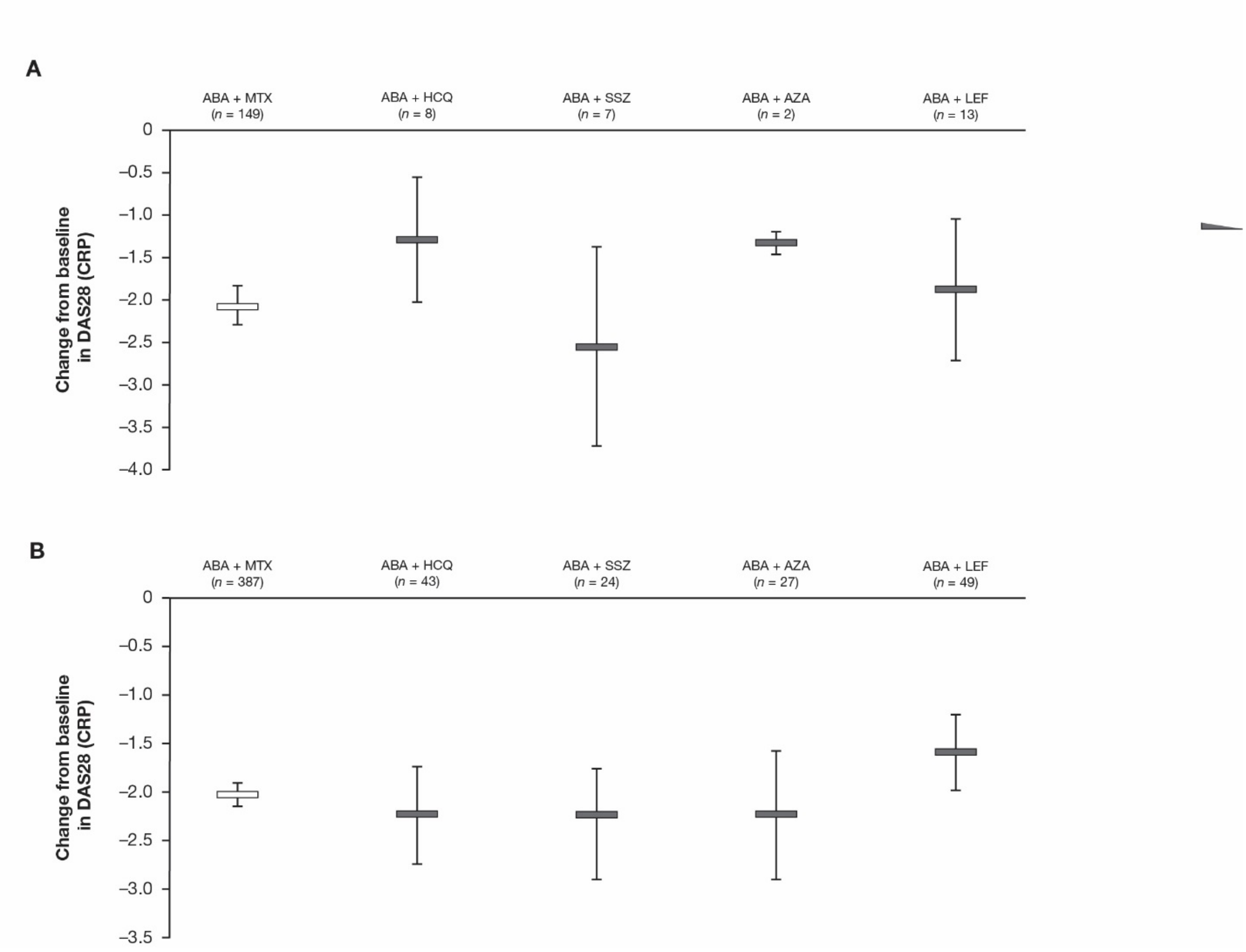

Supplement: Supplementary file 2 — is a figure showing mean change from baseline in DAS28 (CRP) at 6 months in response to abatacept administered in combination with one csDMARD from the (a) ATTAIN and (b) ARRIVE studies (PDF 101 kb) [file 13075_2017_1488_MOESM2_ESM.pdf]
