# Supplementary material for: Abatacept used in combination with non-methotrexate disease-modifying antirheumatic drugs: a descriptive analysis of data from interventional trials and the real-world setting
Source: Arthritis Res Ther. 2018 Jan 2;20:1. doi: 10.1186/s13075-017-1488-5 (PMC5795278; doi:10.1186/s13075-017-1488-5)
Supplement: Supplementary file 3 — is a table presenting safety profile data of csDMARDs administered in combination with abatacept in the ATTAIN, ASSURE and ARRIVE studies (PDF 35 kb) [file 13075_2017_1488_MOESM3_ESM.pdf]

Additional file 3

Supplementary Table 1. Safety of csDMARDs administered in combination with abatacept in the ATTAIN, ASSURE and ARRIVE studies

	ATTAIN					
	**ABA + MTX (n = 175)**	**ABA + HCQ (n = 11)**	**ABA + SSZ (n = 10)**	**ABA + AZA (n = 4)**	**ABA + LEF (n = 17)**	**ABA + all non-MTX csDMARDs (n = 42)**
**Deaths**	0	0	1 (10.0)	0	NR	1 (2.4)
**SAEs**	16 (9.1)	1 (9.1)	2 (20.0)	2 (50.0)	NR	5 (11.9)
**Related SAEs**	4 (2.3)	0	0	1 (25.0)	NR	1 (2.4)
**Discontinuations due to SAEs**	3 (1.7)	0	2 (20.0)	2 (50.0)	NR	4 (9.5)
**AEs**	139 (79.4)	6 (54.5)	7 (70.0)	3 (75.0)	14 (82.4)	30 (71.4)
**Related AEs**	73 (41.7)	4 (36.4)	4 (40.0)	1 (25.0)	6 (35.3)	15 (35.7)
**Discontinuations due to AEs**	5 (2.9)	0	2 (20.0)	2 (50.0)	NR	4 (9.5)
AEs of interest (system organ class)						

**Infections and infestations**	67 (38.3)	3 (27.3)	1 (10.0)	2 (50.0)	11 (64.7)	17 (40.5)
**Skin and subcutaneous tissue disorders**	27 (15.4)	1 (9.1)	2 (20.0)	1 (25.0)	3 (17.6)	7 (16.7)
**Eye disorders**	8 (4.6)	0	1 (10.0)	0	1 (5.9)	2 (4.8)
**Cardiac disorders**	7 (4.0)	1 (9.1)	3 (30.0)	0	1 (5.9)	5 (11.9)
**Blood and lymphatic system disorders**	2 (1.1)	0	1 (10.0)	1 (25.0)	1 (5.9)	3 (7.1)
**Immune system disorders**	2 (1.1)	1 (9.1)	0	0	–	1 (2.4)
**Neoplasms (benign, malignant, and unspecified)**	3 (1.7)	0	1 (10.0)	1 (25.0)	–	2 (4.8)

ASSURE

	**ABA + MTX (n = 535)**	**ABA + HCQ (n = 35)**	**ABA + SSZ (n = 36)**	**ABA + AZA (n = 10)**	**ABA + LEF (n = 62)**	**ABA + all non-MTX**

						csDMARDs (n = 143)
**Deaths**	3 (0.6)	0	0	0	1 (1.6)	1 (0.7)
**SAEs**	54 (10.1)	4 (11.4)	4 (11.1)	1 (10.0)	15 (24.2)	24 (16.8)
**Related SAEs**	9 (1.7)	0	1 (2.8)	1 (10.0)	4 (6.5)	6 (4.2)
**Discontinuations due to SAEs**	12 (2.2)	0	0	0	4 (6.5)	4 (2.8)
**AEs**	480 (89.7)	32 (91.4)	31 (86.1)	8 (80.0)	56 (90.3)	127 (88.8)
**Related AEs**	299 (55.9)	20 (57.1)	21 (58.3)	5 (50.0)	37 (59.7)	83 (58.0)
**Discontinuations due to AEs**	28 (5.2)	0	2 (5.6)	0	6 (9.7)	8 (5.6)
AEs of interest (system organ class)						
**Infections and infestations**	292 (54.6)	24 (68.6)	17 (47.2)	5 (50.0)	44 (71.0)	90 (62.9)
**Skin and subcutaneous tissue disorders**	133 (24.9)	5 (14.3)	9 (25.0)	3 (30.0)	20 (32.3)	37 (25.9)
**Eye disorders**	45 (8.4)	4 (11.4)	4 (11.1)	3 (30.0)	11 (17.7)	22 (15.4)

**Cardiac disorders**	28 (5.2)	1 (2.9)	3 (8.3)	0	7 (11.3)	11 (7.7)
**Blood and lymphatic system disorders**	28 (5.2)	2 (5.7)	4 (11.1)	1 (10.0)	3 (4.8)	10 (7.0)
**Immune system disorders**	13 (2.4)	0	1 (2.8)	0	4 (6.5)	5 (3.5)
**Neoplasms (benign, malignant, and unspecified)**	20 (3.7)	2 (5.7)	0	0	1 (1.6)	3 (2.1)

			**ARRIVE**			
	**ABA + MTX (n = 490)**	**ABA + HCQ (n = 49)**	**ABA + SSZ (n = 32)**	**ABA + AZA (n = 30)**	**ABA + LEF (n = 65)**	**ABA + all non-MTX csDMARDs (n = 176)**
**Deaths**	1 (0.2)	0	0	0	0	0
**SAEs**	45 (9.2)	5 (10.2)	3 (9.4)	2 (6.7)	3 (4.6)	13 (7.4)
**Related SAEs**	14 (2.9)	0	0	0	1 (1.5)	1 (0.6)

**Discontinuations due to SAEs**	7 (1.4)	0	0	0	1 (1.5)	1 (0.6)
**AEs**	383 (78.2)	39 (79.6)	20 (62.5)	27 (90.0)	45 (69.2)	131 (74.4)
**Related AEs**	217 (44.3)	19 (38.8)	9 (28.1)	14 (46.7)	25 (38.5)	67 (38.1)
**Discontinuations due to AEs**	20 (4.1)	2 (4.1)	1 (3.1)	1 (3.3)	3 (4.6)	7 (4.0)
AEs of interest (system organ class)						
**Infections and infestations**	203 (41.4)	14 (28.6)	8 (25.0)	14 (46.7)	27 (41.5)	63 (35.8)
**Skin and subcutaneous tissue disorders**	61 (12.4)	8 (16.3)	2 (6.3)	8 (26.7)	6 (9.2)	24 (13.6)
**Eye disorders**	24 (4.9)	3 (6.1)	0	0	3 (4.6)	6 (3.4)
**Cardiac disorders**	16 (3.3)	0	3 (9.4)	1 (3.3)	1 (1.5)	5 (2.8)
**Blood and lymphatic system disorders**	10 (2.0)	0	2 (6.3)	0	1 (1.5)	3 (1.7)
**Immune system disorders**	7 (1.4)	0	1 (3.1)	1 (3.3)	2 (3.1)	4 (2.3)

**Neoplasms benign, malignant and unspecified**	6 (1.2)	0	0	0	2 (3.1)	2 (1.1)

All data are n (%)

ABA, abatacept; AE, adverse event; AZA, azathioprine; csDMARD, conventional synthetic disease-modifying antirheumatic drug; HCQ, hydroxychloroquine; LEF, leflunomide; MTX, methotrexate; NR, not reported; SAE, serious adverse event; SSZ sulfasalazine.
